# Source attribution of human campylobacteriosis at the point of exposure by combining comparative exposure assessment and subtype comparison based on comparative genomic fingerprinting

**DOI:** 10.1371/journal.pone.0183790

**Published:** 2017-08-24

**Authors:** André Ravel, Matt Hurst, Nicoleta Petrica, Julie David, Steven K. Mutschall, Katarina Pintar, Eduardo N. Taboada, Frank Pollari

**Affiliations:** 1 Groupe de recherche en épidémiologie des zoonoses et santé publique, Faculté de médecine vétérinaire, Université de Montréal, Saint-Hyacinthe, Québec, Canada; 2 Département de pathologie et microbiologie, Faculté de médecine vétérinaire, Université de Montréal, Saint-Hyacinthe, Québec, Canada; 3 Centre for Food-borne, Environmental and Zoonotic Infectious Diseases, Public Health Agency of Canada, Guelph, Ontario, Canada; 4 National Microbiology Laboratory at Lethbridge, Public Health Agency of Canada, Lethbridge, Alberta, Canada; Massey University, NEW ZEALAND

## Abstract

Human campylobacteriosis is a common zoonosis with a significant burden in many countries. Its prevention is difficult because humans can be exposed to *Campylobacter* through various exposures: foodborne, waterborne or by contact with animals. This study aimed at attributing campylobacteriosis to sources at the point of exposure. It combined comparative exposure assessment and microbial subtype comparison with subtypes defined by comparative genomic fingerprinting (CGF). It used isolates from clinical cases and from eight potential exposure sources (chicken, cattle and pig manure, retail chicken, beef, pork and turkey meat, and surface water) collected within a single sentinel site of an integrated surveillance system for enteric pathogens in Canada. Overall, 1518 non-human isolates and 250 isolates from domestically-acquired human cases were subtyped and their subtype profiles analyzed for source attribution using two attribution models modified to include exposure. Exposure values were obtained from a concurrent comparative exposure assessment study undertaken in the same area. Based on CGF profiles, attribution was possible for 198 (79%) human cases. Both models provide comparable figures: chicken meat was the most important source (65–69% of attributable cases) whereas exposure to cattle (manure) ranked second (14–19% of attributable cases), the other sources being minor (including beef meat). In comparison with other attributions conducted at the point of production, the study highlights the fact that *Campylobacter* transmission from cattle to humans is rarely meat borne, calling for a closer look at local transmission from cattle to prevent campylobacteriosis, in addition to increasing safety along the chicken supply chain.

## Introduction

*Campylobacter* is the leading bacterial cause of foodborne enteric disease in most developed countries, with an estimated total incidence of 213,749 domestically-acquired cases (90% credible interval 144,288–308,837) per year among the 32,500,000 Canadians in 2006 [1]. This bacterium is present in many animal reservoirs as well as in the water, to which the few outbreaks registered are often related. Case information illustrates that human infections of campylobacteriosis are mostly sporadic, which is what makes direct identification of the source of contamination difficult. The epidemiological studies conducted in the form of case-controls or intervention studies have helped identify poultry as the probable main source of *Campylobacter* [2]. However, there is some evidence that other sources may play a non-negligible role in *Campylobacter* aetiology. Therefore, source attribution, the quantification of the proportion of disease cases linked to potential sources, may prove helpful to determine the relative importance of the *Campylobacter* sources and hence direct public health efforts more efficiently [3, 4].

The microbial subtyping attribution approaches are currently one promising method to perform source attribution. They are data intensive and best suited to use data from a well-designed integrated surveillance system with an efficient (discriminatory, reproducible), standardised and systematically applied typing method [5]. The data on human cases are to be compared to the data on the sources, hence the necessity of a harmonised typing method to ensure comparability of those data. Moreover, representative isolates from all possible sources of human cases should be available to get reliable results. The data on the sources (reservoirs or vehicles) should thus be based on a representative sampling and cover as many sources as possible [5, 6]. There are two main sorts of microbial subtyping attribution models, the frequency matched attribution models, based on the comparison of human strain types and the distribution of those types in the sources, and the population genetic models based on modelling the organism’s evolutionary history. The frequency matched attribution models require a typing method that has sufficient discrimination while maintaining several types that are in both human cases and sources [3].

Human illness attribution can be undertaken at various points along the transmission route from the reservoirs to the humans, and is usually performed at the point of production (i.e. animal reservoir), point of distribution and point of exposure (i.e. consumption of contaminated food or unintended ingestion of the pathogen with water or because of unhygienic practice) [3, 4]. Most previous studies on *Campylobacter* or *Salmonella* source attribution based on subtype comparison were undertaken at the production point [5–9] and a few at the distribution point [10]. Some studies mixed subtype data of isolates collected in live animals or at slaughter with data of isolates detected at the distribution point (e.g. imported meat) [8].

Comparative exposure assessment is another approach to estimate human illness attribution. It has been more rarely undertaken with only two published studies on *Campylobacter* attribution to our knowledge [11, 12]. Combining the two approaches (comparative exposure assessment and subtyping comparison) was judged worthwhile because it allows one to inform risk management and prioritisation of control strategies for each of the different routes of the pathogen transmission instead of focusing on the reservoir [3].

The subtyping of *Campylobacter*, beyond the species level, for such studies is a challenge. The most widely used method so far is multi-locus sequence typing (MLST), which is highly discriminatory, reproducible and produces easy-to-interpret results [13]. This method has been used in several *Campylobacter* source attribution studies so far including in New Zealand [7, 14], and in various parts of Europe [8, 15–20]. As it is costly and time consuming, routine use of MLST at the scale required for integrated surveillance is impractical; a Dutch research team, for instance, has showed that the use of non-local data from far away countries may result in some geographical bias in the attribution estimates [21].

A new typing method, referred to as comparative genomic fingerprinting (CGF), was recently developed in Canada. This molecular method detects the presence/absence of 40 specific genes that target genetic variability in accessory genome content. This method has been shown to be highly concordant with MLST while having much greater discriminatory power [22]. Moreover, because it is a high-throughput, low-cost, and high-resolution method, CGF is amenable to deployment in a surveillance context [23]. Therefore, the CGF method could be a valuable alternative to MLST, especially in the frame of source attribution based on subtypes.

Finally, this study analyses data from FoodNet Canada, a comprehensive sentinel-based integrated surveillance system for enteric diseases in Canada. It was implemented to understand which sources are contributing to human diseases and to identify risk factor information that can be used to guide more effective interventions for food and water safety. This system collects both microbial and epidemiologic data (e.g. human/animal species, location, and for the human cases, travel information, outbreak implicated information, exposure information, etc.). Among other pathogens, it includes *Campylobacter* isolates from reported human cases, from foods at retail, from farm animals and from surface water within the same region (sentinel site). Within its first sentinel site, the Region of Waterloo, Ontario, a food flow analysis estimated that between 1% and 10% of the beef, pork, and poultry meat available at retail come from local farms [24]. In this context, food animal and their products at retail can be considered independent sources of *Campylobacter* for humans. This setting provided a unique standardized microbial and epidemiological dataset over a well-defined and narrow spatio-temporal frame, thus uniquely fulfilling data recommendations for source attribution by subtyping comparisons [3, 4], while allowing work at the point of exposure.

The study aims to derive source attribution estimates for human campylobacteriosis at the point of exposure using the subtyping comparison approach applied to *Campylobacter* subtyped thanks to CGF combined with the results of a comparative exposure assessment. A secondary objective was to explore the impact of the mathematical modelling on the source attribution estimates and parameters.

## Material & methods

### Sources of isolates

FoodNet Canada’s enhanced human surveillance component collects information on enteric disease cases in its sentinel sites from the local public health authorities. For this paper, *Campylobacter* data from the Region of Waterloo (ROW) sentinel site was analyzed. The ROW is located in southwestern Ontario, Canada and is composed of three urban municipalities and four rural townships with a total population of about 500,000. FoodNet Canada uses the existing laboratory-based surveillance system for reportable diseases as it is mandatory for clinical laboratories to report each case of reportable disease to the local public health authority. FoodNet Canada has enhanced this passive reporting system by implementing a standardized questionnaire on all cases of enteric disease that are reported to the local public health authority. This questionnaire includes detailed risk factor and exposure information (see in [25]. Laboratory results are consolidated with the questionnaire information by the public health authorities, who ultimately provide depersonalized epidemiological and microbiological data to FoodNet Canada. Ethics approval for the surveillance data collection was obtained through the Region of Waterloo Public Health Ethics Review Committee in 2005.

Active source surveillance is an integral part of FoodNet Canada; its three components test samples of uncooked retail meats, manure from local food animal producers, and untreated surface water from five points within the Grand River watershed [26]. Retail meats tested for *Campylobacter* include ground beef, chicken (chicken breast, ground chicken, and chicken nuggets), ground turkey, and pork chops. Food animal manures sampled include beef and dairy cattle, broiler chickens, and swine.

The human clinical isolates included 249 sporadic, domestically-acquired cases and one outbreak-related case randomly chosen among the 22 cases the outbreak encompassed. The selection of only one out of the 22 cases was chosen to avoid any bias towards the source that would be the cause of this outbreak.

Eight potential sources of *Campylobacter* were used: three reservoir (cattle manure, chicken manure, swine manure), four food vehicle (beef meat, chicken meat, pork meat, turkey meat) and one environmental vehicle (surface water).

Samples analyzed during the years 2006 to 2011 inclusively were used to achieve a sufficient number of isolates for rarer sources and some sources that were not sampled uniformly over time (Table 1).

**Table 1:** Distribution of *Campylobacter* isolates with CGF data by year and by origin available for source attribution.

Origin of isolate	2006	2007	2008	2009	2010	2011	Total
Human case	54	52	39	24	36	45	250
Cattle manure	43	24	142	146	160	109	624
Chicken manure	0	0	9	6	7	5	27
Swine manure	11	5	69	75	92	78	330
Beef meat	0	1	0	1	1	0	3
Chicken meat	45	91	52	91	74	93	446
Pork meat	0	2	0	1	3	0	6
Turkey meat	0	0	0	0	2	24	26
Surface water	4	0	0	23	12	13	52
Total	157	175	311	367	387	367	1764

### CGF analysis

*Campylobacter* isolates (n = 1,764) were retrieved from frozen glycerol stocks and subcultured onto blood agar plates under microaerophilic conditions at 42°C for 24–48 hours. Biomass was harvested for DNA extraction using the Qiagen EZ1 BioRobot, or EZ1 Advanced XL with the Qiagen Blood and Tissue Kit according to manufacturer’s instructions (Qiagen, Mississauga, Canada). CGF40 PCRs were performed as described previously [22]. PCR products were analyzed using the QIAxcel capillary electrophoresis system with the DNA Screening Kit. The 40 PCR targets were scored as binary data based on presence or absence of bands of expected size, which were then compiled to create a CGF pattern. CGF subtypes are routinely defined based on pattern similarity of isolates in the CGF database at three similarity thresholds (90%, 95% and 100%) and based on hierarchical position in the clustered database. Only the 100% similarity threshold was used to define CGF subtypes in this study (S1 File).

### Data analysis

The number of different CGF subtypes were calculated for each origin (human cases or potential sources) and overall. Within each origin, the number of subtypes specific to this origin and the subtypes also found in other origins (common subtype) were tabulated. The subtypes found among human isolates and shared with a single source were also identified.

### Source attribution estimations

Three methods, falling under the general subtyping comparison approach, were applied to the *Campylobacter* isolates characterized by their CGF subtype to calculate source attribution estimates: the proportional similarity index, the Dutch model, and the Hald model [8, 14, 27]. The models and their equations are explained in the following paragraphs. For sake of clarity and consistency between the methods’ equations, we define several parameters in a manner that is in alignment with previous papers as much as possible. They are:

h_*i*_ = number of human cases with subtype *i*
b_*ij*_ = the number of *Campylobacter* positive isolates for subtype *i* and source *j* (no restriction is made on whether there are human cases with subtype *i* or not)
r_*ij*_ = b_*ij*_ / ∑_*i*_ b_*ij*_, the proportion of *Campylobacter*-positive isolates of subtype *i* in source *j*
s_*j*_ = the number of samples testing negative for *Campylobacter* in source *j*
p_*ij*_ = b_*ij*_ / [(∑_*i*_ b_*ij*_)+s_*j*_], the proportion of *Campylobacter*-positive samples of subtype *i* in source *j*, referred to as prevalence

#### Proportional similarity index

The proportional similarity index (PSI) was used to measure the degree of overlap of the frequency distributions of CGF subtypes between the human isolates and a group of non-human isolates [27]. It was computed as follows:

$${PSI}_{j}=1-0.5\sum_{i}\left|h_{i}/\sum_{i}h_{i}–r_{ij}\right|,\;\mathrm{for}\;a\;\mathrm{given}\;\mathrm{source}\;j$$ (1)

with h_*i*_ and r_*ij*_ as previously defined. PSI_j_ ranges from zero to one, where one indicates that the two groups are identical and zero means they have no types in common. 95% confidence intervals were computed using bias-corrected and accelerated non-parametric bootstrap, as implemented in Stata (StataCorp. 2013. Stata: Release 13. Statistical Software. College Station, TX) using the bootstrap command with the bca option. The bootstrap command was applied to a custom estimation program that calculates the PSI (available on request).

#### Dutch model

Source attribution estimates based on the Dutch model were computed as follows [28]:

$$\lambda_{ij}=\frac{k_{ij}}{\sum_{j}k_{ij}}\;h_{i}$$ (2)

where k_*ij*_ is the relative occurrence of subtype *i* in source *j*, a parameter explained later, and h_*i*_ as explained earlier.

λ_*ij*_ is the estimated number of cases of subtype *i* from source *j*. The sum across subtypes gave the total number of cases from source *j*, λ_j_:

$$\lambda_{j}=\sum_{i}\lambda_{ij}$$ (3)

Final source attribution proportions, z_*j*_ were then calculated (Eq 4). Confidence intervals were calculated using the same custom estimation program discussed for the PSI. Hypothesis tests for differences between attribution estimates also used this program and are based on the same bootstrap method.

$$z_{j}=\frac{\lambda_{j}}{\sum_{j}\lambda_{j}}$$ (4)

#### Hald model

The model was adapted from the *Campylobacter* attribution model by Boysen et al. [8], which is an adaptation of a *Salmonella* attribution model [29]. It uses a Bayesian framework to estimate the number of human cases attributed to the various sources under study. It is described according to the following equations:

$$\lambda_{ij}=k_{ij}\times q_{\text{i}}\times a_{\text{j}}$$ (5)

where λ_*ij*_ is the expected number of cases of subtype *i* from source *j*, k_*ij*_ is the occurrence of subtype *i* in source *j* and is defined below, q_*i*_ is the subtype-dependent parameter (interpreted as the specific ability of subtype *i* to cause disease), and a_*j*_ the source-dependent parameter (interpreted as the specific ability of source *j* to transfer the pathogen to humans). The q_*i*_ and a_*j*_ are unknown. These parameters were set with a hierarchical prior [8] and an exponential prior distribution, respectively. The log of q_*i*_ was set to follow a normal distribution N(0, *τ*) with the prior distribution for *τ* being the Gamma(0.01, 0.01) distribution as suggested by Mullner [7]. The prior for a_*j*_ was assigned an Exponential (0.02, 0.02), also suggested by Mullner [7]. Following previous work [30], the value for each q_i_ “anchor” was set to fixed value for each subtype that was source-specific, meaning those subtypes *i* that were present from human isolates and found in only one source. In those cases the q_*i*_ value was:

$$q_{i}=\frac{h_{i}/\sum h_{i}}{k_{ij}}$$ (6)

The models were estimated using OpenBugs (http://www.openbugs.net/w/FrontPage), with 5000 burn-in iterations followed by 40,000 iterations.

#### Defining the occurrence k_ij_

For the primary objective of source attribution at the point of exposure, we set the k_ij_ parameter to be r_ij_*E_j_, where r_ij_ was defined above and E_j_ is the exposure value for source j. The exposure value E_j_ for the eight sources tested were derived from a concurrent *Campylobacter* comparative exposure assessment study [12]. This work estimated the average population exposure of *Campylobacter* to humans (measured in terms of number of organisms ingested/person/day) from 13 different sources including seven of the eight sources of interest here. It was conducted for the province of Ontario that includes the area from which the surveillance data used in this study originate. The E_j_ values for chicken, beef and pork retail meat was directly taken from the comparative exposure assessment study results. The exposure for the turkey meat was adapted from the chicken meat by using the relevant parameter values whenever available (i.e. turkey net consumption, retail ground turkey contamination rate for *Campylobacter*). The exposure value for contact with animals was available from the exposure assessment study for all food animals together and for exposure from either living on a farm or visiting a farm. Thanks to a previous survey of healthy humans on their behavior and exposure in the same area [31], we were able to breakdown the estimated value of exposure through food animals by living on a farm for the three animal species of interest (chicken, cattle and swine) proportionally to the species that people living on farm have contact with and similarly for the exposure through visiting a farm. Finally the values for exposure through living on a farm and through visiting a farm were summed up by animal species. The exposure values used in this study as E_*j*_ are presented in Table 2.

**Table 2:** Exposure value used in the exposure-based attribution models [12].

Route	E_j_ value*	90% probability interval	Data source
**Chicken manure**	1.54x10^-2^	[1.34x10^-3^; 4.21x10^-2^]	The value of exposure through contact with food animals was derived from a comparative exposure assessment (ref) and was proportioned to chicken based on the frequency of living on property with chicken or visiting a farm with chicken that were derived from a healthy people survey conducted exactly in the same area in 2009–2010 [31]
**Cattle manure**	2.98x10^-2^	[2.57x10^-3^; 8.13x10^-2^]	Value of exposure through contact with food animals was derived from a comparative exposure assessment [12] and was proportioned to cattle based on the frequency of living on property with cattle or visiting a farm with cattle that were derived from a healthy people survey conducted exactly in the same area in 2009–2010 [31]
**Swine manure**	5.01x10^-6^	[1.88x10^-4^; 5.91x10^-3^]	Value of exposure through contact with food animals was derived from a comparative exposure assessment [12] and was proportioned to swine based on the frequency of living on property with pigs or visiting a farm with pigs that were derived from a healthy people survey conducted exactly in the same area in 2009–2010 [31]
**Beef meat**	2.40 x10^-3^	[1.24 x10^-4^; 7.21 x10^-3^]	Directly derived from a comparative exposure assessment [12]
**Chicken meat**	2.07 x10^-1^	[2.17 x10^-3^; 8.96 x10^-2^]	Directly derived from a comparative exposure assessment [12]
**Pork meat**	1.17 x10^-3^	[9.19 x10^-5^; 3.5 x10^-3^]	Directly derived from a comparative exposure assessment [12]
**Turkey meat**	9.95 x10^-3^	[1.30 x10^-3^; 4.43 x10^-2^]	Generated running the chicken exposure model used in the comparative exposure assessment (Pintar, … Ravel, Risk Analysis accepted) with turkey specific data for consumption, portion size, *Campylobacter* contamination (prevalence and load in meat)
**Surface water**	8.27x10^-3^	[7.49 x10^-4^; 2.03 x10^-2^]	Directly derived from a comparative exposure assessment [12]

* CFU ingested/person/day

Because of uncertainty in the exposure values, a sensitivity analysis was undertaken for the exposure-based Dutch model to assess to what extent the source attribution values estimated by the model change when the exposure value for a given source is increased or decreased, the values for all other parameters being kept constant. Probability intervals for exposure estimates varied between 1 and 2 log from the mean estimate to include all possibilities, even extreme scenarios. Based on this, we used sensitivity inputs that are -2, -1.5, -1, -0.5, 0, 0.5, 1, 1.5 and 2 (log_10_ units) times the mean value to explore the association between exposure values E_j_ and source attribution estimates λ_*j*_.

For the secondary objective of exploring the impact of including exposure values, we set the k_ij_ parameters to be r_ij_ (proportion-based model) and p_ij_ (prevalence-based model), ran the Dutch and the Hald model accordingly and visually compared the outcomes. We also explore the impact of the structure of the Hald model on the source-dependent parameters (a_*j*_) and subtype-dependent parameters (q_*i*_). The a_*j*_ parameters are supposed to be source specific, implying a unique value for a given source. The original Hald model designed for attribution at the point of production included a food consumption parameter M_j_ [29]. Later studies using this model dropped the consumption parameter on the basis that the changes in the model structure (with or without the food consumption) are absorbed by the a_j_ parameters [7, 32, 33]. The evidences for such absorption is limited [7]. Furthermore, recent studies proposed or used a modified Hald model where its structure includes another parameter: the proportion of food consumed raw or undercooked [33]. In order to better document the absorption of the changes in the model structure by the a_j_ parameters, we had the posterior distribution of k_ij_*a_*j*_ computed when running the three Hald models and looked at the correlation between their mean values. Our hypothesis was that the value of k_ij_*a_*j*_ would be identical or at least very close if the absorption effect mentioned held true. We also computed the relative change in their mean values between the three models using the prevalence-based model as the reference. Finally, the Hald model assumes the existence of subtype-dependent parameters (q_*i*_). According to this, the q_*i*_ values for a given subtype should be the same independently of the model structure. We explored this assumption by looking at the correlation between the proportion, prevalence and exposure-based model in their posterior mean values.

## Results

### Description of samples, isolates and subtypes

Table 3 describes the isolates available from the different origins as well as their prevalence in the potential sources as derived from the FoodNet Canada active surveillance. Overall, 453 different CGF subtypes were found among the 1768 isolates analyzed. The number of isolates per subtype ranged from 1 to 71. Just over eighty percent of all subtypes (81.2%, 368/453) were unique to an origin (Table 3). Unique subtypes were found among all sampling origins except for chicken manure and beef meat.

**Table 3:** Distribution of samples, isolates and CGF subtypes by sampling origin.

	Human cases	Chicken manure	Cattle manure	Swine manure	Chicken meat	Beef meat	Pork meat	Turkey meat	Surface water	Total
**# isolates**	250	27	624	330	446	3	6	26	52	1768
**# samples**	Not applicable	427	1023	602	1575	901	901	172	588	6439
**Prevalence**	Not applicable	6.3%	61.0%	54.8%	28.3%	0.3%	0.7%	15.1%	8.8%	
**# CGF subtypes**	104	10	135	128	166	3	6	17	36	453*
**# subtypes unique to the origin (# isolates)**	48 (53)	0 (0)	85 (127)	109 (232)	94 (124)	0 (0)	2 (2)	5 (5)	23 (30)	368* (569)
**% unique subtypes**	46.2	0.0	62.9	85.1	56.6	0.0	33.3	29.4	63.9	81.2
**# subtypes common to other origins (# isolates)**	56 (197)	10 (27)	50 (497)	19 (98)	72 (322)	3 (3)	4 (4)	12 (21)	13 (22)	85 * (1199)
**Distribution of subtypes (isolates) common to** **:										
**Human cases**	-	9 (25)	33 (398)	5 (27)	49 (279)	3 (3)	4 (4)	10 (17)	7 (9)	
**Chicken manure**	9 (57)	-	5 (39)	0 (0)	8 (81)	0 (0)	0 (0)	1 (3)	1 (2)	
**Cattle manure**	33 (157)	4 (9)	-	12 (86)	39 (214)	3 (3)	4 (4)	5 (12)	9 (15)	
**Swine manure**	5 (13)	0 (0)	12 (96)	-	17 (47)	0 (0)	1 (1)	0 (0)	2 (2)	
**Chicken meat**	49 (187)	8 (24)	39 (416)	17 (92)	-	3 (3)	4 (4)	11 (20)	9 (14)	
**Beef meat**	3 (19)	0 (0)	3 (51)	0 (0)	3 (6)	-	0 (0)	0 (0)	0 (0)	
**Pork meat**	4 (15)	0 (0)	4 (34)	1 (22)	4 (29)	0 (0)	-	1 (2)	1 (1)	
**Turkey meat**	10 (38)	1 (1)	5 (39)	0 (0)	11 (111)	0 (0)	1 (1)	-	3 (5)	
**Surface water**	7 (28)	1 (1)	9 (115)	2 (7)	9 (84)	0 (0)	1 (1)	3 (10)	-	

* different from the sum of the row because some subtypes were present in more than one origin

** the number of subtypes and of isolates within a column exceeds the number of common subtypes and isolates found in an origin because the some subtypes were present in more than one other origin

The 250 human isolates were distributed among 104 CGF subtypes, 48 of these subtypes (53 isolates) being unique to the human cases, and the remaining 56 subtypes (197 isolates) being found in at least one of the studied sources, most often among chicken meat (49 subtypes encompassing 187 human isolates) and cattle manure (33 subtypes encompassing 157 human isolates) (Table 2). Sixteen subtypes encompassing 23 human isolates were present in only a single source: one subtype was common to chicken manure, four to cattle manure, 17 to chicken meat, and one to surface water.

Ten subtypes were found in chicken manure, none being unique, and were most often found in human cases (9 subtypes) and chicken meat (8 subtypes), and one subtype each in turkey meat and surface water (Table 3). The 624 cattle manure isolates were distributed among 135 CGF subtypes with 85 being unique to this source and 50 subtypes (497 isolates) being found in all the other sources including human cases (33 subtypes) and chicken meat (39 subtypes). The 330 swine manure isolates were grouped into 128 subtypes, 109 being unique, and 19 (98 isolates) were found in human cases (5 subtypes) and in other studied sources, mostly chicken meat (17 subtypes) and cattle manure (12 subtypes), but not in chicken manure, beef meat and turkey meat.

The 446 chicken meat isolates fell into 166 subtypes, 94 unique and the other 72 (322 isolates) covering all of the other origins studied, mostly among human cases (49 subtypes) and cattle manure (39 subtypes). The three beef meat isolates were of three different subtypes that were also common to human cases, cattle manure and chicken meat: The six pork meat isolates were each of a different subtype, two were unique to pork meat whereas the other four isolates were present in human cases, cattle manure and chicken meat. Turkey meat isolates were of 17 different subtypes, five unique and 12 (21 isolates) being found in the other origins, most often in chicken meat (11 subtypes) and human cases (10 subtypes), but not in swine manure and beef meat. About two-thirds of the subtypes found in surface water were unique (23 subtypes) whereas the other third were found in all the other origins except beef meat, and more frequently in chicken meat (nine subtypes), cattle manure (eight subtypes), and human cases (seven subtypes).

The CGF subtypes among the chicken meat (PSI = 0.421) and the cattle manure (PSI = 0.356) were moderately similar to the human subtypes, whereas the similarity with human isolates was low for surface water (PSI = 0.110), beef meat (PSI = 0.076), pork meat (PSI = 0.060), and swine manure (PSI = 0.019) (Table 4).

**Table 4:** Proportional Similarity Index (PSI) between human campylobacteriosis isolates and isolates from potential sources based on their Comparative Genomic Fingerprinting subtypes.

Source	PSI	95% Confidence Interval	
Chicken manure	0.216	0.097	0.336
Cattle manure	0.356	0.284	0.428
Swine manure	0.019	0.004	0.034
Chicken meat	0.421	0.304	0.537
Beef meat	0.076	0.005	0.147
Pork meat	0.060	0.000	0.121
Turkey meat	0.152	0.077	0.227
Surface water	0.110	0.029	0.190

### Attribution at the point of exposure

**Fig 1:**
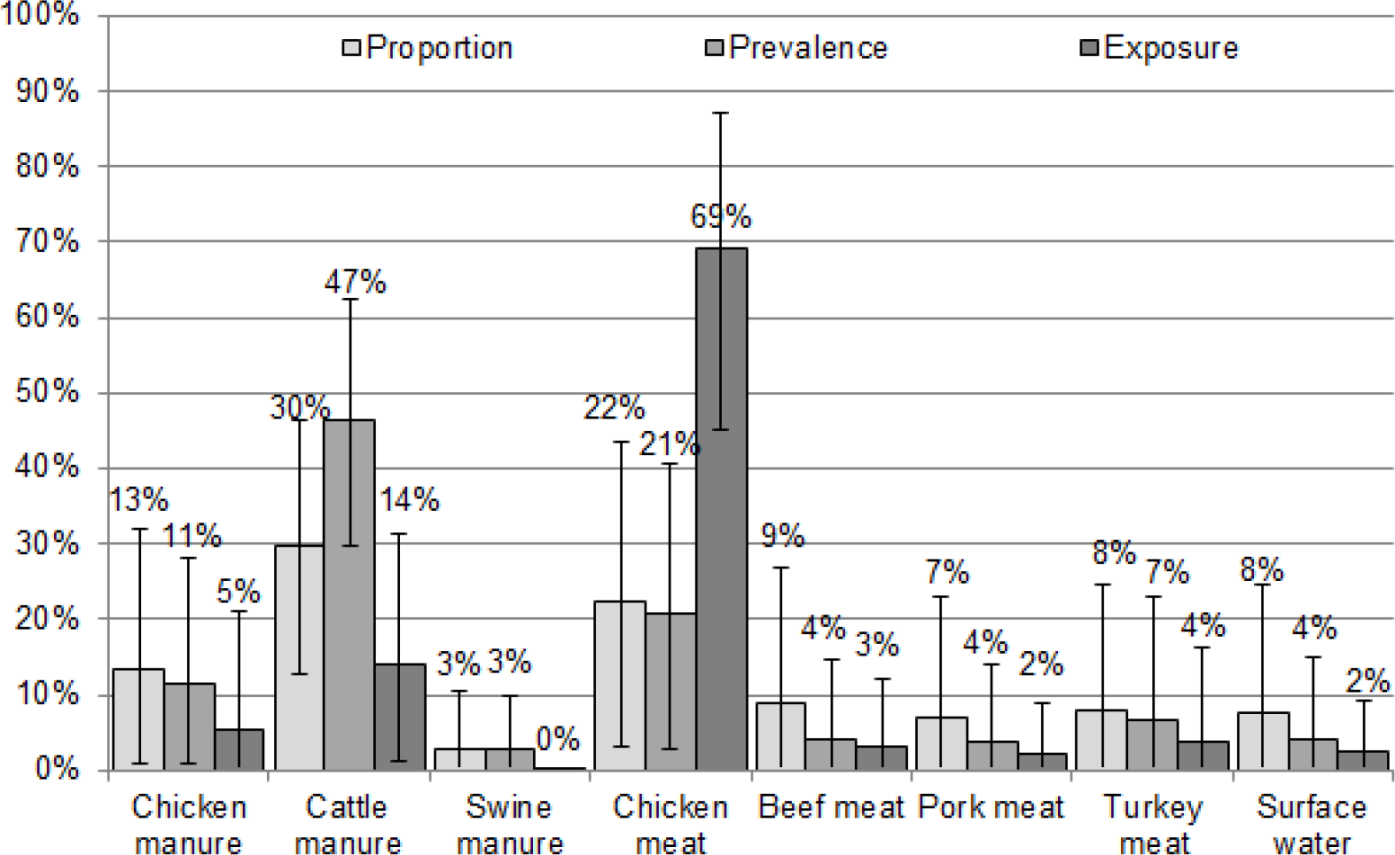
Source attribution results (estimated proportion of human campylobacteriosis cases attributed to each source for 197 attributable cases) with 95% credible interval according to the proportion-, prevalence- and exposure-based Hald models.

The exposure-based Hald model runs converged without any issues and the posterior mean number of cases by subtype closely matched the actual number of cases by less than one case for the majority of the subtypes. Chicken meat was by far the main source of human cases of campylobacteriosis (69%) followed by cattle manure (14%). Cases were almost uniformly spread across the remaining sources, except for swine manure (0.02%), with attribution estimates ranging between 2 and 5% (Fig 1).

**Fig 2:**
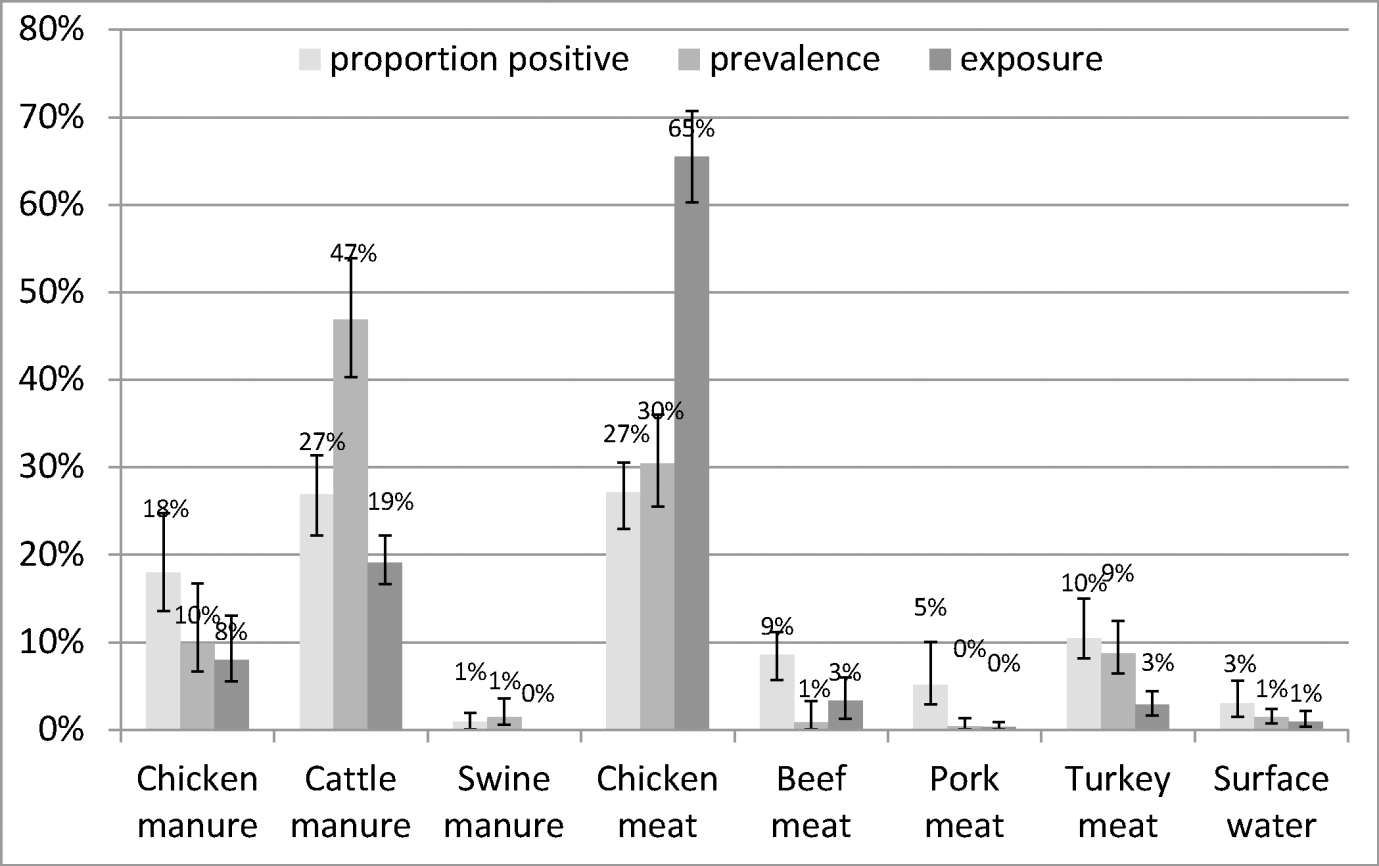
Source attribution results (estimated proportion of human campylobacteriosis cases attributed to each source for 197 attributable cases) with 95% confidence interval according to the proportion-, prevalence- and exposure-based Dutch models.

According to the Dutch exposure-based model, chicken meat was by far the largest contributor to human cases at 65%, with cattle manure coming in second at 19%, chicken manure third at 8.0%, followed by the small contributors of beef meat (3.3%), turkey meat (2.9%), surface water (0.9%), pork meat (0.3%), and swine manure (0.0%) (Fig 2).

**Fig 3:**
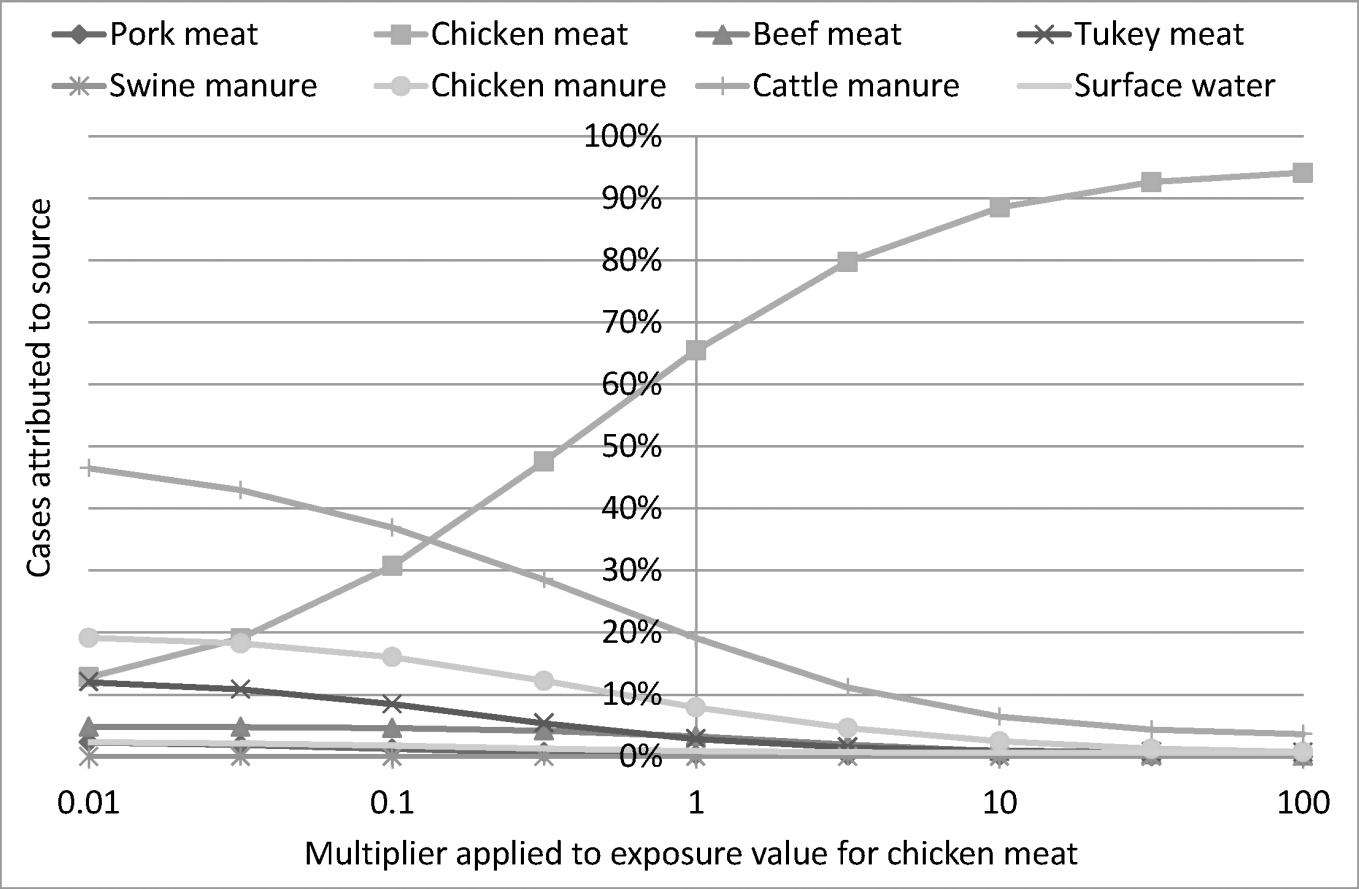
Results of the sensitivity analysis of the exposure-based Dutch model for chicken meat.

The sensitivity analysis undertaken on the exposure-based Dutch model showed that the proportion of human cases attributed to each source varied greatly from changes in the exposure estimates (E*i*). Varying the chicken meat exposure value only from 0.01 times its mean value to 100 times changed the percentage of cases attributed to chicken meat from 13% to 94%, with cattle manure losing the most (see Fig 3 for chicken meat). Varying cattle manure exposure values only by these amounts produced the second greatest change in attributed values: 2.4% to 69% with the vast majority of this increase coming from losses in chicken meat. The third greatest change was in chicken manure, changing from 0.8% to 27%. Chicken meat lost the most from this change, with cattle manure changing very little. Turkey meat was next, varying from 0.0% to 18% with most of this coming from chicken meat. The remaining sources varied less than 10%.

### Comparing results from the proportion-, prevalence-, and exposure-based models

All proportion- and prevalence-based Hald model runs converged without any issues and the posterior mean number of cases by subtype closely matched the actual number of cases by less than one case for the majority of the subtypes. The outcomes of the three proportion-, prevalence-, and exposure-based models were different, especially for the latter one (Fig 1). The proportion-based model showed that cattle manure was the primary source of campylobacteriosis (30%), with chicken meat (22%) and chicken manure (13%) coming next, then the meats (beef meat (8.9%), turkey meat (7.9%), and pork meat (7.1%)) and surface water (7.7%) while 2.9% cases were attributed to swine manure. Results for the prevalence-based model indicated that cattle manure was the primary source of campylobacteriosis (47%), with chicken meat (21%), chicken manure (11%) coming next, then turkey meat (6.8%) while 2–5% were attributed to swine manure, pork meat, beef meat and surface water each.

Using the Dutch model, important changes in attribution were also observed between the exposure-, proportion- and prevalence-based models (Fig 2). Results for the proportion-based model indicated that chicken meat, chicken manure and cattle manure were the primary sources of campylobacteriosis, and to a lesser extent turkey, beef and pork meat. Chicken meat (27%) and cattle manure (27%) contributed equally with chicken manure a close third at 18%. The prevalence-based model results indicated cattle manure was the largest contributor at 47%, chicken meat second at 30% and chicken manure third at 10%.

### Impact of the Hald model structure on the a and q parameters

Forty q_*i*_ parameters (56 subtypes– 12 for which the value was fixed by the data) were estimated by each proportion, prevalence-, and exposure-based Hald model. Their posterior mean values had the same range, between 0.5 and 4, for the three models. The mean values estimated through the proportion-based model were highly correlated with the prevalence-based model (r = 0.96) and exposure-based model (r = 0.72) estimates (S1 Fig). The mean of the posterior values for a_*j*_ varied between Hald models with correlation of 0.90 between proportion- and prevalence-based models, of 0.31 between proportion- and exposure-based models, and of 0.12 between prevalence- and exposure-based models (S2 Fig). The sets of posterior mean values of k_ij_*a_*j*_ showed two distinct and strong correlation patterns between the proportion- and the prevalence-based models (S3 Fig). One correlation was apparent for some k_ij_*a_*j*_ values between the exposure- and the prevalence-based model, whereas a lack of correlation was shown for the other values (S4 Fig). Finally, the k_ij_*a_*j*_ mean values changed significantly between the models (S5 Fig). The relative changes were similar within each source between the exposure- and the prevalence-based models with the exception of the chicken meat where a large variation in the k_ij_*a_*j*_ values was observed. The relative changes in the k_ij_*a_*j*_ values were high between and within sources with the exception of the chicken meat.

## Discussion

This study includes several original and innovative features. It aimed at providing source attribution estimates for human campylobacteriosis at the point of exposure, whereas most studies have been undertaking at the point of production or distribution. It also combined two source attribution approaches: the comparative exposure assessment and the microbial subtyping comparison. This combination of approaches has been suggested [3] but nothing similar has been published to our knowledge. For the microbial subtyping comparison, we use the Comparative Genotype Fingerprinting method to define the subtypes based on the presence or absence of 40 genes [22]. Finally, another strength and novelty was the quality and comprehensiveness of the isolates and data used: the isolates came from a comprehensive sentinel site surveillance system encompassing enhanced surveillance of human clinical cases and active surveillance in food animal, retail meat and surface water within the same geographic area (http://www.phac-aspc.gc.ca/foodnetcanada/index-eng.php). This allowed for a focus on domestically-acquired cases of campylobacteriosis, along with the ability to capture subtypes at the reservoir level (through isolates from chicken, beef and dairy cattle, and swine manure samples), at the vehicle level (through isolates from chicken, turkey, beef and pork retail meat) and from surface water. All the surveillance isolates used for this study came from the same geographic area, thus reducing the likely bias introduced when using disperse geographic origins of isolates [21]. Similarly for time, the effort in source sampling and testing was intense enough that all isolates used came from the same, relatively small period of time. As a result, the isolates and data available for attribution greatly fulfilled the requirement for microbial subtyping comparison [3, 4].

### Campylobacteriosis attribution at the point of exposure

According to this study, chicken meat is by far the most important source of human campylobacteriosis (accounting for two-third of the attributable cases) whereas exposure to cattle is the second source (accounting for 14–19% of attributable cases), the other sources (live chicken, live pigs, chicken, turkey, and pork meat, surface water) being minor sources of campylobacteriosis. The particular design and settings of this study limit its direct comparability with other studies on microbial subtype comparison for source attribution undertaken on a larger (usually national) scale and at the point of production or distribution. Nevertheless, their general findings agree with ours about the very first importance of the chicken commodity with bovine source ranking second [8, 14, 17, 21, 34–36]. Our main findings are consistent with the findings of previous Canadian studies on campylobacteriosis attribution, most having used approaches other than subtyping comparison or comparative exposure assessment [37–42].

### Importance of proximity to cattle

With its focus on point of exposure, the present study is valuable for detangling the importance of various pathways in bringing *Campylobacter* form its natural reservoirs to humans. While other studies with a point of production focus found that cattle is the second important reservoir for campylobacteriosis, our findings specify that the transmission from cattle is merely not meat borne but it seems more related to proximity with live animals. In another Canadian study, 25.8% of *C*. *jejuni* infection cases were attributed to cattle (fecal samples), second after chicken meat (64.5%) based on Bayesian clustering method that used multilocus subtypes [42]. The authors hypothesized that unpasteurized milk consumption may explain this attribution to cattle as raw milk and unpasteurized dairy products have been identified as the cause of several campylobacteriosis outbreaks (for example [43] or [44] and as a risk factor for sporadic infection [2]. Another hypothesis is the local contamination of water sources and wells in rural area by cattle manure whereby people may be contaminated locally [45–48], in contrast to a wider spread of *Campylobacter* through the watershed that our water samples would have detected. These results should encourage public health authorities, food safety and water quality stakeholders to target the cattle farms for the prevention of *Campylobacter* transmission to humans through contact with cattle, consumption of raw milk or well water contaminated or not adequately treated, in addition to continue their efforts to reduce *Campylobacter* contamination along the poultry supply chain.

### Importance of water as a source of Campylobacter

The study deals with surface water as an exposure for humans to Campylobacter considering that this water is used as recreational water at some places within the study area and is the intake of drinking water system for a large part of the population. Our attribution estimates for water (<5%) is lower than the results of a study that attributed 7.4% of cases to water in another region of Canada based on Bayesian clustering method that used multilocus subtypes [42], and than the 9% of campylobacteriosis being waterborne according to a Canadian expert elicitation study [40]. The watershed from which our samples were taken was found regularly contaminated by *Campylobacter* according to a microbial water contamination study, with some contamination coming from waterfowl [49]. Most CGF profiles found in water were unique to water and few were common to humans or to beef, chicken or pigs manure (Table 3) implying that this water is little impacted by animal production activities or human wastes in comparison with what has been found in other studies [50]. It also implies that if contamination by waterfowl or other wild animals occurs, it does not necessarily lead to many human cases as observed elsewhere [51]. Considering our results attributing relatively more campylobacteriosis cases to cattle than to surface water and the discussion above, we concluded that the attribution to water should include isolates from a more representative sampling of water, notably covering small and individual sources of drinking water.

### The value of CGF for source attribution purpose

This study was the second to use the new method of Comparative Genotype Fingerprinting to define subtypes of *Campylobacter* as the basis of the comparison between human cases and the investigated sources. Using CGF, Deckert et al. showed that urban campylobacteriosis cases were more likely to be infected by *Campylobacter* strains found in retail chicken compared to rural cases [52]. Previous studies on source attribution based on subtypes comparison have used species, antimicrobial resistance, MLST, PFGE and other typing alone or in combination to define subtypes and derive their source attribution estimates [17, 34, 35, 42, 53–56]. Our study showed that CGF is an alternative method. CGF defined enough subtypes among the clinical isolates and among the source isolates, some being unique but most being shared with at least one other origin, so that microbial source comparison can be performed. We used the 100% similarity threshold across the 40 genes to define CGF subtypes. Two more relaxed thresholds (95% and 90%) were tested on the three Dutch models and the attribution results agreed quite closely across the thresholds indicating some robustness of the attribution results to the CGF subtypes definition. Because the CGF method tests for the presence or absence of 40 genes, it does not provide as detailed genotype information as Multilocus Sequence Typing, hence it is not suitable for attributing cases to source based on detailed phylogenetic differences as performed by the asymmetric island attribution method.

### Relevance of the exposure-based model structure

Both the Hald and Dutch models modified with the inclusion of exposure yielded similar results, increasing our confidence in the findings and in the model structure. Obviously, the inclusion of exposure in any model makes a large difference in the attribution estimates, hence it should be highly recommended to include exposure value (E_j_) whenever attributing source at the point of exposure is the purpose. Estimating exposure to *Campylobacter* or any enteric zoonotic pathogens however is a difficult endeavour facing several uncertainties resulting in wide confidence interval for the exposure estimates [11, 12]. Nevertheless, even an imprecise measurement of exposures is worthwhile to enter in any exposure-based model because the large differences in exposure (in terms of a few to several log units) between the sources will still be reflected in the attribution estimates, as shown by our sensitivity analysis. This may differ when modelling attribution at the production or distribution point. The inclusion of food consumption as a parameter was part of the original Hald model [29]; the addition of another parameter, the proportion of food consumed raw or undercooked for each source, was proposed and has been used [33, 57]. Whether to include or not these two parameters in the Dutch or Hald models is not that clear with some studies providing evidence that they are not necessary for the Hald model whereas other studies used one (food consumption) or the two. Noteworthy, the consideration for these two parameters is aligned with the concept of exposure, at least for any foodborne exposure. Actually, the comparative exposure assessment study from which we used some results included food (portion) consumption and the impact of any treatment that may reduce the pathogen (e.g. cooking), among its variables to define exposure. This exposure assessment went further down in including other variables (e.g. pathogen concentration, cross-contamination) to define and quantify exposure and dealt with non-foodborne exposure in a similar, structured and systematic way. We consider the exposure assessment model used as a generalization of the ways for taking into account the relative weight of sources that have been proposed (including food consumption and the proportion of food consumed raw or undercooked) in previous models, going further in detail by including more parameters and being expendable to none foodborne exposure. For example, it allows dealing with exposure to pets or wild birds, two under looked exposures to *Campylobacter*, something that we could not perform because of lack of isolates from these two sources.

### Hald model structure and parameters

We explored the validity of some features of the Hald model. First, the model includes a subtype-specific parameter (q_i_) that should be constant independently of the model structure [8, 29]. Our results provided empirical evidence for the validity of such subtype-specific parameter. Second, it was reasoned that the food consumption parameter that was part of the original Hald model formula was not a necessity and could be dropped from the model without impact on attribution estimates [7]. It was explained by the fact that the value of the source-specific parameter would change and absorb the lack of the consumption value in the model. The impact of including or not the food consumption parameter was formally tested in the context of human salmonellosis attribution [33]. This test showed that the attribution estimates were similar with or without food consumption when using the Hald model, but different when using the Dutch model. Our results contrast with this findings since our attribution estimates varied between the proportion-, the prevalence-, and the exposure-based models for both the Dutch and the Hald approaches. Furthermore, our assessment of the k_ij_*a_*j*_ quantity, that should be similar over the three models if the absorption phenomenon exists, showed that this absorption is plausible between the proportion and the prevalence-based models, but is less likely true for the exposure-based-model. It should be noted that the exposure values we used for the sources varied considerably between the sources, by several units on a log scale, whereas the food consumption values used by Mughini-Gras and Van Pelt were of similar order of magnitude across their four sources (pig, cattle, broilers, layers/eggs): 42.2, 19.2, 17.3, and 13.9 kg/person/year, respectively [33]. The large range among our exposure values might allow for an extreme assessment of the absorption phenomenon compared to the previous study. We conclude that the absorption phenomenon claimed for the Hald model does not hold true for all model structures, and cautions should be exercised when deciding on the model structure.

### Study limitations

The study limitations include the inclusion of all isolates independently of the *Campylobacter* species. This provides a global figure of source attribution of human campylobacteriosis cases (mainly due to *C*. *jejuni* infection), masking probable differences between species as demonstrated elsewhere [17, 35, 56]. The number of isolates usable per source investigated was variable. For two sources (beef and pork meat) this number was below 25, the minimum number of isolates for a given source for a source attribution estimation based on the asymetric island model [21], meaning a reduced capacity (statistical power) in attributing cases to these two sources in our study. On the other hand these low numbers reflected the rare presence of *Campylobacter* in these sources despite intensive sampling. In addition, the study was carried out in a small area of half million people, which precludes the extrapolation to other regions or even nationally because of its specific settings. Other factors that can influence the relative importance of the sources of *Campylobacter* have not been considered and should be in future works, including the patient’s place of residency (rural vs. urban) [34], the patient’s age, and the season [36]. No isolates from pets or wildlife were available from the study area over the time period. Wildlife, especially wild birds, and pets, especially dogs, have been shown to be source of campylobacteriosis [17, 34, 58–61] and should be considered in future attribution endevours. Like previous work, the study does not consider the influence of person-to-person transmission, either directly (human to human) or indirectly (human to food or water to human). Finally, the study considered exposure but it is recognized that exposure does not systematically lead to infection and disease. Such limitations are general and are applicable to all other campylobacteriosis source attribution efforts.

## Conclusion

This study successfully combined comparative exposure assessment and microbial subtype comparison with subtypes defined by comparative genomic fingerprinting to quantify the relative contribution of eight sources of human campylobacteriosis at the point of exposure in a Canadian area that provided high quality data thanks to an integrated surveillance system. It showed that chicken meat is the primary source of human campylobacteriosis, with 65 or 69% (depending on models) of attributable cases assigned to it, followed by live cattle (14 or 19%). Live chicken and pigs, pork, beef, and turkey meat and water are minor sources for human campylobacteriosis. Prevention of human campylobacteriosis should benefit of further studies on the exact contamination pathways between live cattle and humans. Strengthening efforts to improve food safety along the chicken supply chain is critical to reduce the burden of campylobacteriosis in Canada. Attention to the other pathways, particularly contact with animals and water, should be maintained and even reinforced for an overall reduction of the campylobacteriosis burden.

## Supporting information

S1 Fig Correlations between the mean of the posterior q_*i*_ subtype specific parameter values (n = 40 subtypes) estimated by the prevalence-based Hald model and those estimated by the proportion- and the exposure-based Hald models. (TIF)

S2 Fig Correlations between the mean of the posterior a_*j*_ source specific parameter values (n = 8 sources) estimated by the prevalence-based Hald model and those estimated by the proportion- and the exposure-based Hald models. (TIF)

S3 Fig Correlation of the mean of the posterior values of the k_ij_*a_*j*_ values (n = 40 subtypes * 8 sources) estimated by the proportion- and the prevalence-based Hald models. (TIF)

S4 Fig Correlation of the mean of the posterior values of the k_ij_*a_*j*_ values (n = 40 subtypes * 8 sources) estimated by the exposure- and the prevalence-based Hald models. (TIF)

S5 Fig Relative changes by source in the mean of the posterior values of the k_ij_*a_*j*_ (n = 40 subtypes * 8 sources) estimated by the proportion-, the prevalence-, and the exposure-based Hald models. (TIF)

S1 File Campylobacter CGF data analyzed. It includes the isolate identification number, the *Campylobacter* species, the origin of the isolate, the year of the sample collection, the CGF pattern, and the CGF subtype number based on the 100% similarity.
(XLSX)
