# Supplementary figures and images for: Source attribution of human campylobacteriosis at the point of exposure by combining comparative exposure assessment and subtype comparison based on comparative genomic fingerprinting
Source: PLoS One. 2017 Aug 24;12(8):e0183790. doi: 10.1371/journal.pone.0183790 (PMC5570367; doi:10.1371/journal.pone.0183790)

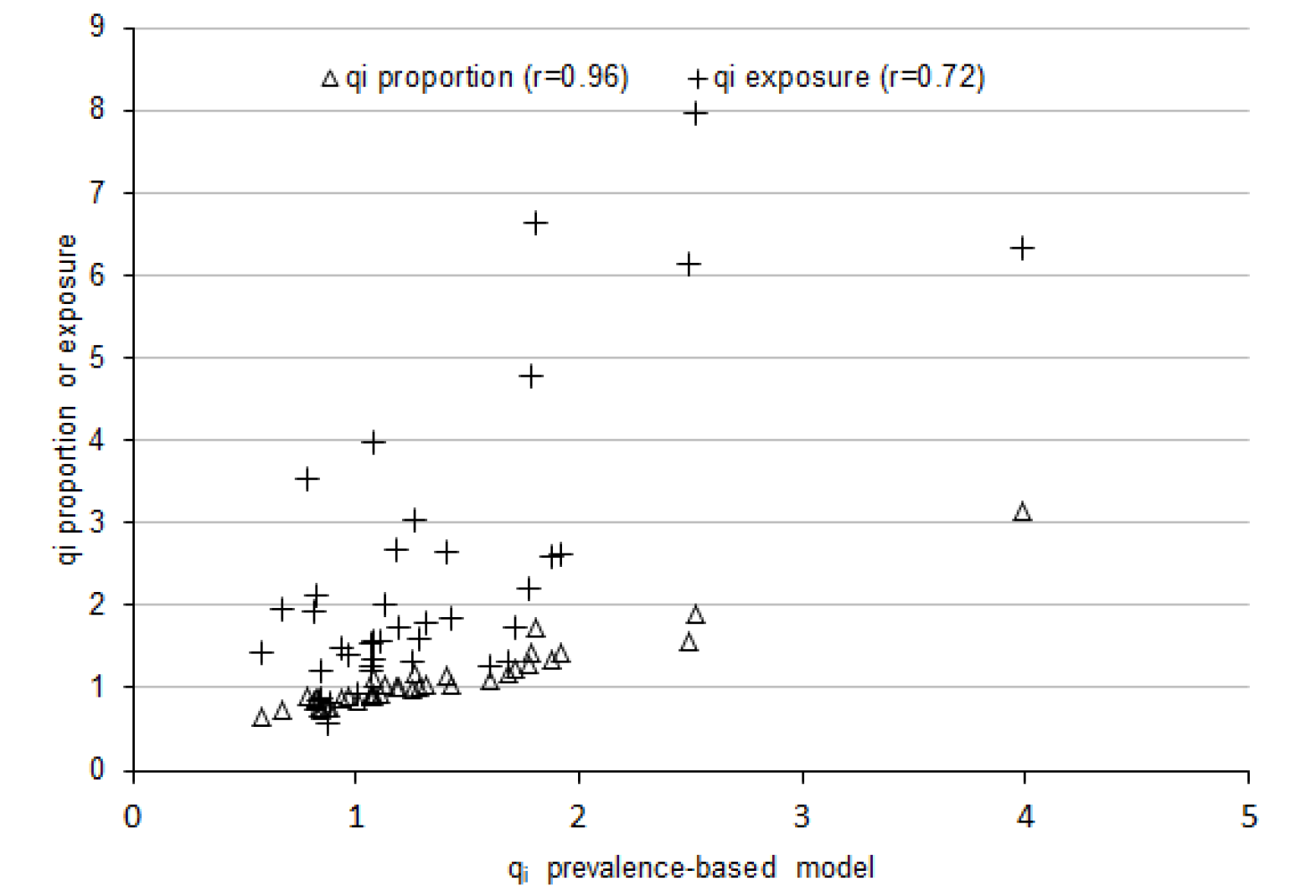

Supplement: S1 Fig — (TIF) [file pone.0183790.s001.tif]

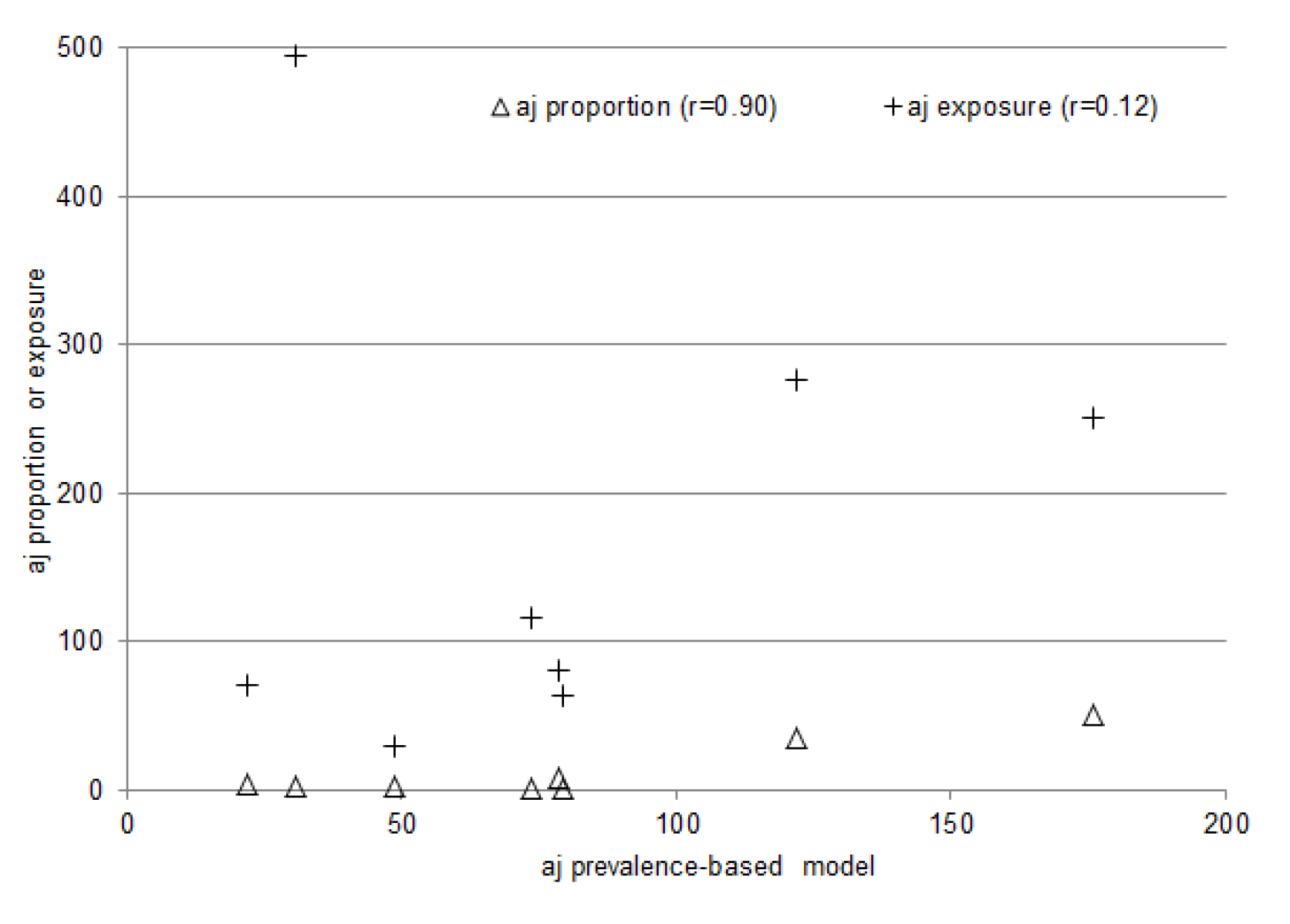

Supplement: S2 Fig — (TIF) [file pone.0183790.s002.tif]

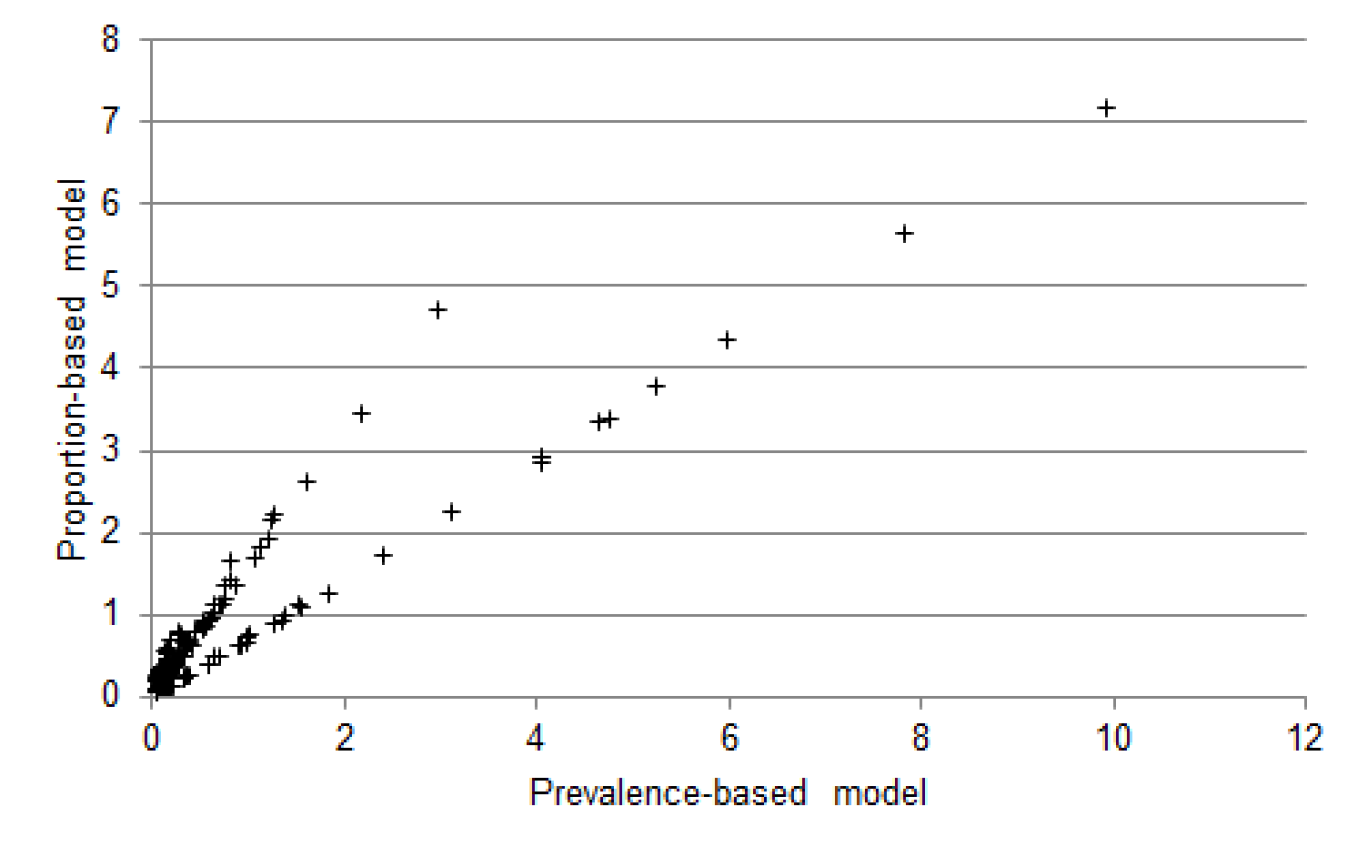

Supplement: S3 Fig — (TIF) [file pone.0183790.s003.tif]

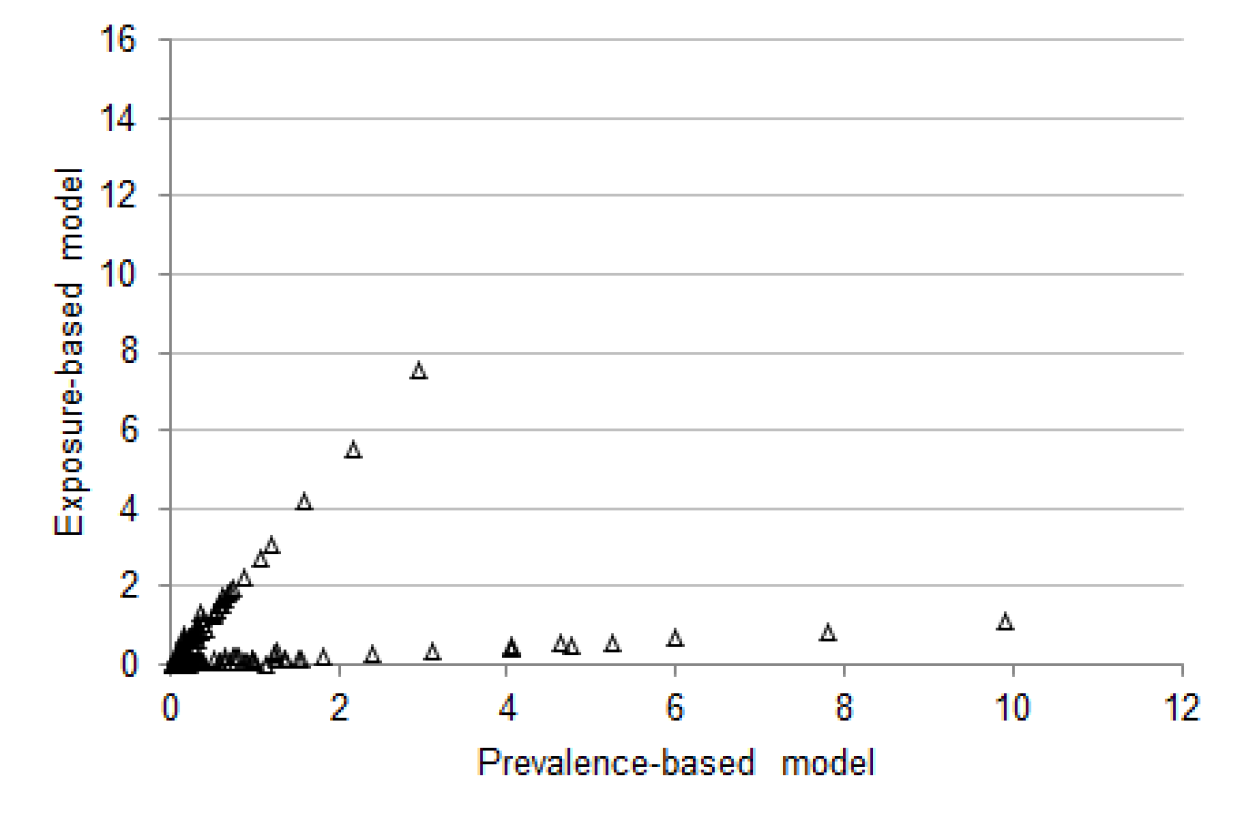

Supplement: S4 Fig — (TIF) [file pone.0183790.s004.tif]

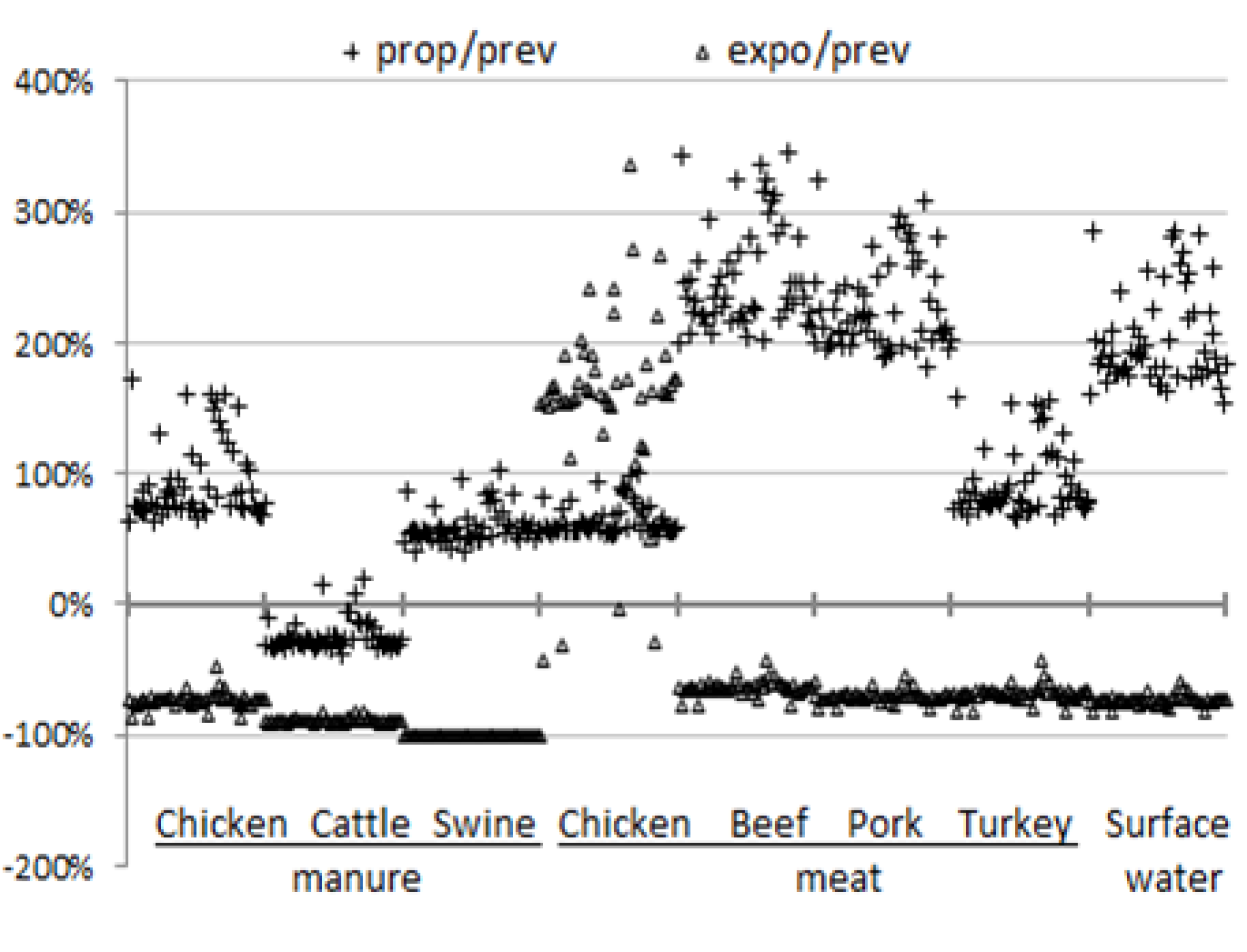

Supplement: S5 Fig — (TIF) [file pone.0183790.s005.tif]
